# Fundamental Dynamical Modes Underlying Human Brain Synchronization

**DOI:** 10.1155/2012/912729

**Published:** 2012-06-28

**Authors:** Catalina Alvarado-Rojas, Michel Le Van Quyen

**Affiliations:** Centre de Recherche de l'Institut du Cerveau et de la Moelle Épinière (CRICM), INSERM, UMRS 975 and CNRS UMR 7225, UPMC, Hôpital de la Pitié-Salpêtrière, 75651 Paris cedex 13, France

## Abstract

Little is known about the long-term dynamics of widely interacting cortical and subcortical networks during the wake-sleep cycle. Using large-scale intracranial recordings of epileptic patients during seizure-free periods, we investigated local- and long-range synchronization between multiple brain regions over several days. For such high-dimensional data, summary information is required for understanding and modelling the underlying dynamics. Here, we suggest that a compact yet useful representation is given by a state space based on the first principal components. Using this representation, we report, with a remarkable similarity across the patients with different locations of electrode placement, that the seemingly complex patterns of brain synchrony during the wake-sleep cycle can be represented by a small number of characteristic dynamic modes. In this space, transitions between behavioral states occur through specific trajectories from one mode to another. These findings suggest that, at a coarse level of temporal resolution, the different brain states are correlated with several dominant synchrony patterns which are successively activated across wake-sleep states.

## 1. Introduction

Although much is known about the functional architecture of the brain, its large-scale dynamics remain poorly understood. At this macroscopic level, the existence of large-scale dynamics is confirmed by numerous functional brain mapping studies, showing that multiple distributed cortical areas coordinate their activities during perceptuomotor behavior [1–3]. Phase synchrony is an important candidate for such large-scale integration, mediated by neuronal groups that oscillate in specific bands and enter into precise phase locking over a limited period of time [4, 5]. It is also known that the coordination of these large-scale patterns is subject to drastic changes in different behavioral states, possibly because of state-dependent shifts in both neuromodulatory balance and the thalamic gating of sensory inputs [6–8]. A major challenge is to determine general principles that govern the spontaneous succession of global brain states across the entire wake-sleep cycle. Recently, using simultaneous local field potentials recorded in multiple forebrain areas in behaving rats, it has been shown that several brain states (e.g., quiet waking, active exploration, and slow-wave sleep) can be represented as distinct clusters in a multidimensional state space representing various levels of local and long distances synchronization [9]. These results suggest that major brain states that comprise the wake-sleep cycle can be identified by a frequency-dependent neuronal cooperativity that involves different oscillatory levels within individual brain regions and transient synchronization across brain areas. Motivated by this study, we investigated the long-term dynamics of human brain synchronization using intracranial recordings of epileptic patients. During the phase of presurgical evaluation, these patients were recorded for up to 7–14 days in order to capture habitual seizures. Invasive EEG recording from intracranial electrodes was required to localize focal epileptic activity and to determine the exact spatial relationships between centers of epileptic activity and functionally significant areas. In contrast to scalp EEGs, intracranial recordings provide, temporally distant from epileptic seizures, episodes of normal brain activity that are highly differentiated, down to millimeter spatial resolution. The good spatial resolution and the high signal-to-noise ratio offered by intracranial electrodes have been proven valuable in the detection of large-scale dynamical relationships between cortical networks, in both the time and frequency domains and allow a reliable separation of local and long-range mechanisms [5, 10].

## 2. Methods

### 2.1. Database

We examined intracranial recordings from 5 subjects with refractory partial epilepsy undergoing presurgical evaluation, hospitalized between February 2002 and July 2007 in the epilepsy unit at the Pitié-Salpêtrière hospital in Paris. Each patient was continuously recorded during several days (duration range, 9–20 days; mean duration, 15 days) with intracranial electrodes (Nicolet acquisition system, CA, USA; 16-bit, bandwidth at 3 dB: 0.1–150 Hz). Signals were digitized at 400 Hz. Depth electrodes were composed of 4 to 10 cylindrical contacts 2.3 mm long, 1 mm in diameter, 10 mm apart center-to-center, mounted on a 1 mm wide flexible plastic probe. Subdural electrodes were strips with 4 to 8 one-sided circular contacts, 2.3 mm in diameter and with a center to center separation of and 10 mm. Pre- and post- implantation MRI scans were evaluated to anatomically and precisely locate each contact along the electrode trajectory. The selection of the sites to implant varied among patients and was made entirely for clinical purposes. Three patients had bilateral depth electrodes placed within the hippocampus in combination with subdural strips added to sample lateral or inferior cortices of the temporal and frontal lobes; two patients had only unilateral depth electrodes in various regions of the neocortex and hippocampus. (Patient 1: 47 channels; unilateral subdural strip electrodes covering the frontal lobe and basal regions of the temporal lobe; unilateral depth electrodes placed in the insula, amygdale, and hippocampus. Patients 2 and 5: 47 and 50 channels; bilateral subdural strip electrodes covering the basal regions of the temporal lobe, lateral, and posterior temporal cortex; bilateral depth electrodes placed in the hippocampus. Patient 3: 32 channels; unilateral depth electrodes placed in the basal regions of the temporal lobe, lateral temporal cortex, and frontal lobe. Patient 4: 38 channels; unilateral depth electrodes placed in basal regions of the temporal lobe, lateral temporal cortex, insula, and amygdala; bilateral depth electrodes placed in the hippocampus). An epileptologist visually evaluated the EEG recordings. Electrodes that exhibited interictal epileptiform discharges (i.e., clearly distinguishable spikes, sharp waves, or spike-and-waves complexes) were identified and were removed from the analysis. Waking and sleep stages were determined by video monitoring and confirmed by the visual inspection of the corresponding EEG recordings. Sleep was here mostly defined as nonrapid eye movement (NREM) periods by the presence of K-complexes (stage 2) or slow waves (stage 3-4).

### 2.2. Large-Scale Synchronization Analysis

The analysis of phase synchronization between neuronal signals was introduced by [11] to overcome some limitations of conventional methods which cannot disentangle instantaneous amplitudes and phases [10]. The term “synchronization” is used in its strict sense, as a statistical measure of the degree to which two signals are phase locked during a short-time period. Recent studies have demonstrated the ability of this measure to discriminate transient synchronization in intracranial EEG data [12]. Our analysis followed several steps: first, signals from nonoverlapping, consecutive 5-second periods were filtered with a bandpass corresponding to a particular frequency component. Second, the instantaneous phase of each filtered window was extracted by means of the Hilbert transform. Third, the degree of phase locking between a pair of EEG channels was quantified by the trial average of the phase differences on the unit circle in the complex plane:

(1) $$\text{PLV}=||\frac{1}{n}\overset{n}{\underset{1}{\sum }}e^{i[\phi_{1}(t)-\phi_{2}(t)]}||,$$

where *n* is the number of data points in each time window. This phase-locking value (PLV) varies between 0 (independent signals) and 1 (constant phase lag between the two signals).

**Principal Components Analysis**

PCA is a method for identifying patterns in data of high dimension and expressing that data in such a way as to highlight their similarities and differences. Since patterns can be hard to find in data of dimension greater than three, where the luxury of graphical representation is not available, PCA is a powerful tool for “visualizing” and “compressing” that data, by reducing the number of dimensions, without much loss of information. PCA usually starts with a large number of data or “observation” vectors, with as many components as system variables. The methods of linear algebra then allow the selection of a special, ordered set of basis vectors, the so-called “principal components.” These vectors are of unit (Euclidean) length and are mutually orthogonal, with pairwise “dot products” equal to zero. The first principal component vector represents a single axis in space. When you project each observation vector onto that axis, the resulting values form a new variable. And the variance of this variable is the maximum among all possible choices of the first axis. The second principal component represents another axis in space, perpendicular to the first. Projecting the observations onto this axis generates another new variable, whose variance is the next largest among all possible choices of this second axis, and so forth. The full set of principal component vectors contains as many elements as the number of original variables. But it is common for the sum of the variances of the first few principal components to exceed, say, 80–90% of the total variance of the original data. By examining plots of these few new variables, researchers often develop a deeper understanding of the driving forces that generated the original data. In mathematical terms, the unordered set of principal component vectors is simply the set of eigenvectors of the covariance matrix of the observation vectors. The first principal component is the eigenvector with the largest eigenvalue, the second principal component corresponds to the next largest eigenvalue, and so on.

**Hierarchical Clustering**

Hierarchical clustering uses a so-called “agglomerative” approach, in which individual items, perhaps patterns, are joined to form larger groups. These groups are then joined again and again, until the process has been carried to completion, forming a single hierarchical “tree.” This hierarchical clustering proceeds in a simple manner from an initial state, in which each cluster consists of a single item. First, having selected a metric or distance function *d*, the matrix of distances between all possible pairs (*r*, *s*) of clusters (*d*(*r*, *s*)) is formed, and the two closest (or “most similar”) clusters are chosen. This is the first true stage in the “clustering” process. If several pairs of clusters have the same separation distance, a predetermined rule is used to decide between alternatives. Second, the two selected clusters are merged to produce a new, larger cluster. Third, the distances are calculated between this new cluster and all remaining clusters. Fourth, this process continues iteratively, until a single cluster, consisting of all the individual items, remains. Whenever it is necessary to determine the two “closest”, most similar clusters, Ward's method is used: for every cluster pair (*r*, *s*), the sum of the squares of the distances between all items in their composite cluster and the mean (or centroid) of that cluster is computed [13]. The pair which achieves the minimum of this measure is then selected as the pair to be combined, thus maximizing within-cluster homogeneity. Spurious, small sized clusters were removed. The optimum grouping was defined as the one that minimized the ratio between intracluster and intercluster distance measures, producing the used value for the expected number of clusters. Several other distance measures between clusters (e.g., average, centroid, median distances) were tested with qualitatively similar results. We use the implementations from the Matlab's Statistics Toolbox (The Mathworks, Natick, MA).

**State-Space Representation**

**Figure 1:**
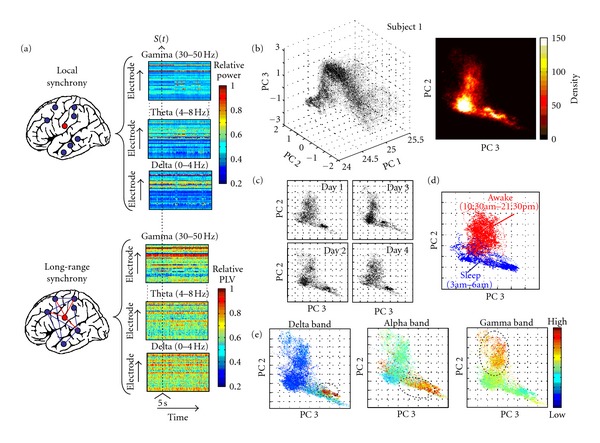
After a narrow band filtering of the intracranial EEGs in the delta (1–4 Hz), theta (4–8 Hz), alpha (8–13 Hz), beta 1 (13–20 Hz), beta 2 (20–30 Hz), and gamma (30–50 Hz) frequency bands, local- and long-range synchronizations were, respectively, estimated by the spectral power of each recording contact and by the mean phase-locking values (PLV) between every contact and all the others. This computation allows the characterization of the multifrequency synchronization patterns of each time window *t* as a vector *S(t)*. (b) Scatter and density plots of 4 successive days (i.e., 96 hours, patient 1), in the space of the first principal components. (c, d) Distributions in the state space across several days and during waking and sleep states. (e) The spectral amplitudes were color coded in the state space, characterizing three main internal frequencies of individual regions in the delta, alpha, and gamma bands.

Following previous work [12], consecutive 5-second EEG recording periods were quantified by a synchronization vector (*S*) that characterizes the multifrequency synchronization patterns in the delta (0.5–4 Hz), theta (4–8 Hz), alpha (8–13 Hz), beta 1 (13–20 Hz), beta 2 (20–30 Hz), and gamma (30–50 Hz) frequency bands. Higher frequency bands >50 Hz were not considered here because the corresponding activities mainly reflect short-lasting (<100 msec) and low-voltage events, having only a small implication in average synchronizations computed over windows of a few seconds. For every window of time and every frequency band, *S* quantified two different types of synchronization [5] (Figure 1(a)): (i) local synchronization, estimated by the spectral power of each recording contact and reflecting the frequency-specific summation of coherent currents in a sufficient number of cells that generate externally detectable local field potentials [5] and (ii) long-range synchronization, estimated by the phase-locking values (PLV) for every possible combination of different contacts, characterizing the temporal relationships between different brain regions in a particular frequency band [11]. On the basis of all pairwise computations, we computed for each contact the average synchronization, defined as the mean PLV between a given contact and all the others. Furthermore, separately for each frequency band, all the values of local and long-range synchronization were normalized by the minimum and maximum over all the recording contacts to produce normalized values bounded between 0 and 1. This normalization factor is used to adjust the data to compensate for experimental variability and to “balance” the values from the local and long-range synchronizations being compared. For 50 channels, the dimension of *S* is 50 × 6 × 2 = 600, we typically analyzed ~5 × 10^4^ time windows (2–4 days). To reduce this high-dimensional and possibly redundant data to a lower dimensional space and to reveal hidden underlying factors controlling the dynamics, we used the principal components analysis (PCA). PCA of *S* led to the determination of the underlying modes that characterize the network states, rank-ordered by their importance, allowing the representation of these states as distinct clusters in a low-dimensional state space [9]. The first three principal components (PCs) of *S* were used, typically representing around 90% of the total variance. In order to reduce the small-scale variability and to average over microstructures to yield large-scale temporal structures, resulting PCs were further smoothed with a moving average filter of one minute width.

## 3. Results

We analyzed long-term intracranial recordings of 5 epileptic patients, continuously recorded with a EEG-video monitoring system for successive days (duration ranging from 38 to 103 hours), one day after the implantation of the electrodes and several hours before the first seizure (separated from 10 to 48 hours, depending on the data set). Patients continued taking their standard doses of anticonvulsant medications during this period. The patients had been implanted with both depth electrodes and subdural electrodes distributed over subcortical and neocortical regions. The positioning of electrodes varied among patients (see Section 2.1). We observed that the first three principal components were able to identify and characterize several distinct groups of states across the wake-sleep cycle. While the number of possible synchronization patterns can be very large, we found that most of them occupy an L-shape structure, as can be seen in scatter and density plots (Figure 1(b)). Distributions in the state space were quantitatively similar across several days (Figure 1(c)). Comparative analyses of the behavioral states of the patients showed that the waking and sleep states occupied different regions in the state space (Figure 1(d)). When pooled spectral amplitudes were color coded on this space [9], it was possible to characterize three segregated regions with internal dominant frequencies (Figure 1(e)). The higher gamma power values were observed within a specific region of the state space, mostly associated with the waking state. This observation supports previous reports showing that gamma synchronizations seem to be essential for waking-state information processing. Clearly separated, higher delta powers were exclusively localized in the deep sleep region, also reflecting a maximal distance to the spectral-coded gamma region. Finally, a distinct frequency region in the range of sleep spindles (12–15 Hz, alpha band in Figure 1(e)) was localized near the region associated with delta/slow oscillations [14]. This remarkable frequency segregation of three domains in the state space confirms that distinct synchronization modes mapped different behavioral states [9]. Similar patterns were not observed using only local synchronizations (i.e., spectral powers), suggesting that long-range synchronizations between distant regions are required.

**Figure 2:**
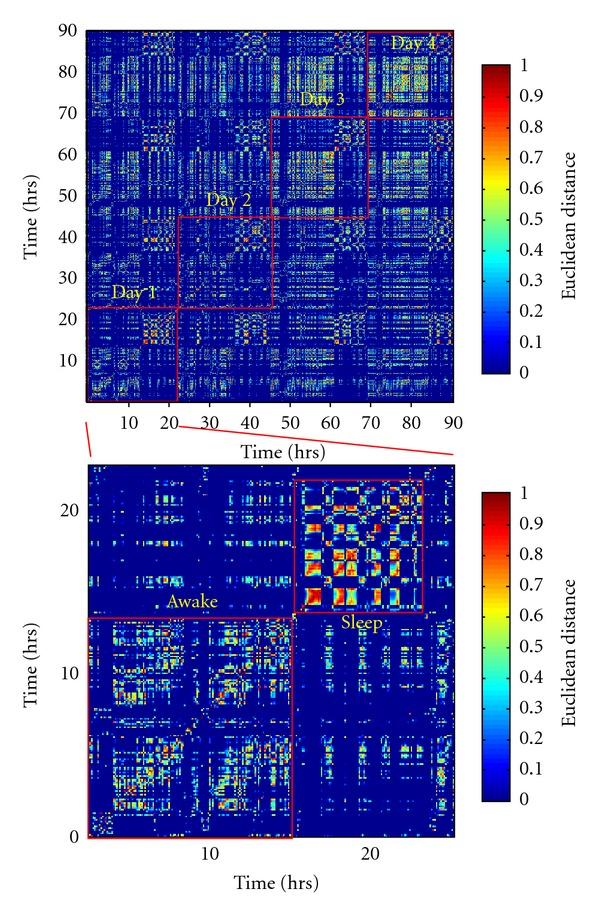
Correlation matrix showing the similarity of all the windows compared with each other over 4 successive days (upper map) and during one day (lower map).

**Figure 3:**
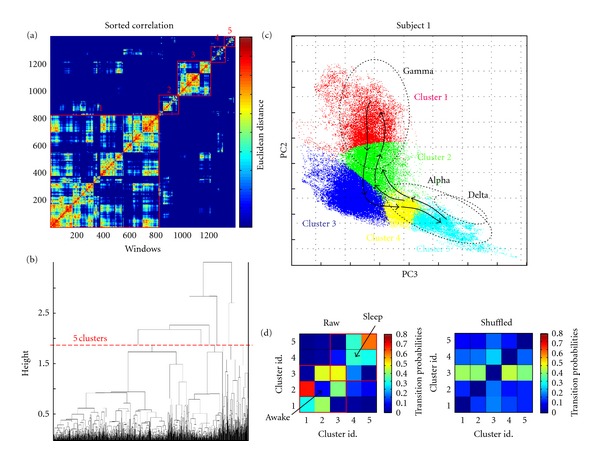
(a) Same correlation matrix as in Figure 2, now sorted in order of similarity by the clustering algorithm. (b) Dendrogram of correlation matrix. Levels of the dendrogram correspond to different sets of clusters in the correlation matrix. At the top of the dendrogram, a single branch signifies that all avalanches are in one cluster. Just below this, the dendrogram divides into two branches, representing a set of two clusters. Branching continues further down the dendrogram until every window is in its own family. The red line crosses the dendrogram at minimum of the ratio between intracluster and intercluster distances and indicated that 5 clusters can be identified (note here that one small sized cluster was removed). The 5 corresponding clusters were reported in the sorted correlation matrix (a). (c) The corresponding 5 clusters were reported in the state space, coded by different colors. The probabilities of transition between the different clusters are depicted using different arrow sizes (small: 0.2 < *P* < 0.4 and large: *P* > 0.4). Direct cluster-to-cluster transitions were mostly identified between proximal modes in the state space. (c) Matrices of transition probabilities between the characteristic modes defined by clustering, for both the actual and shuffled data.

To investigate these recurrent structures more precisely, all the windows were checked against each other for similarity via the euclidian distance in the PC space (Figure 2). Visual inspection of correlation matrices revealed a large number of positive correlations, suggesting that many of the windows produced were recurrent over successive days. Hierarchical clustering algorithms were used to rearrange a correlation matrix from temporal order to order of similarity (Figure 3(a)). When presented in order of similarity, several large squares of high-correlation values appeared on the diagonal of the matrix, suggesting that the windows could be grouped into several clusters that were highly similar within themselves. Within several of the large cluster in a sorted matrix, there were smaller subclusters with even greater correlations (Figure 3(a)). The relationships between these clusters and subclusters could be succinctly described by a dendrogram (Figure 3(d)). Each line that cut across the dendrogram at a different level represented a different way of grouping the windows into clusters. The best clustering was defined at the minimum value of the ratio between intracluster and intercluster distances. We found that most synchronization patterns can be reasonably fit into 3–5 characteristic modes (Figures 3(d) and 3(c)).

**Figure 4:**
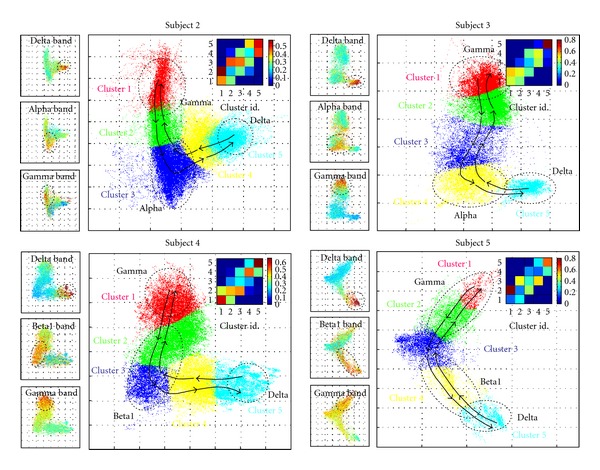
Global state space for 4 patients. For each patient, several characteristic clusters were identified by a hierarchical clustering algorithm and were coded by different colors (analyzed periods for patient 2: 103 hours; patient 3: 38.7 hours; patient 4: 86 hours; patient 5: 40 hours). The probabilities of transition between the different clusters are depicted using different arrow sizes (small: 0.2 < *P* < 0.4 and large: *P* > 0.4). Note that the global dynamic structures governing the trajectories across the state space are similar among all the different patients (insets: matrices of transition probabilities). In a comparable way, the color-coded spectral state spaces conserved three different segregated regions in the delta, alpha/beta, and gamma bands (first column).

To further investigate their global dynamic structures, we explored possible causal relationships by deducing the matrix of transition probabilities between the characteristic modes within a time period under 2 minutes (Figure 3(d)). Concerning the inner stability of individual modes, sleep states represent the most stable dynamics, with the strongest probability to remain in the same mode (*P* ≈ 0.6). In contrast, we observed a significantly greater inner instability for the modes associated with wakefulness (*P* ≈ 0.3). Direct cluster-to-cluster transitions were mostly identified between proximal modes in the state space, those with high probabilities (see the transition matrix in Figure 3(d) and arrows in Figure 3(c) for *P* > 0.2). Direct cluster-to-cluster transitions between nonadjacent modes are rare (*P* < 0.1). Thus the most frequent trajectories are surprisingly simple, showing, on average, a tendency to follow a flow visiting successively adjacent characteristic modes across wake-sleep states. To measure the statistical significance of these dynamical structures, it is necessary to compare the actual data to what would be caused by chance. We used a shuffling to permute the temporal sequence, preserving the original spatial structures in the PC space, but destroying all dynamical structures. Transition matrices of 50 shuffled data were generated, and a maximum matrix was constructed for each patient. As expected, this transition matrix obtained from the shuffled data hinted that preferred cluster-to-cluster transitions between proximal modes could only be found in the actual data (Figure 3(d)). Surprisingly, although a considerable degree of interpatient variability is to be expected from the different electrode implantations, the L-shaped distributions in the state space were quantitatively similar across the five patients (Figure 4). Furthermore, we found a remarkable similarity in the spectral-coded state space, including three segregated regions in the delta, alpha/beta, and gamma bands. Finally, the global dynamic structures governing the trajectories between proximal modes are conserved among the different patients.

## 4. Discussion

Regardless of the relatively small sample size used in the present study, replicability of the results across subjects leads us to believe that global brain states can be mapped into a low-dimensional space based on the degree of local- and long-range synchronization between multiple cortical areas. Automatic cluster analysis made it possible to quantitatively assess the similarities between these different synchronization patterns and to identify functional categories and natural transitions between them. Using this compact representation, we report, with a remarkable similarity across the patients with different locations of electrode placement, that the complex patterns of brain synchrony during the wake-sleep cycle can be represented by a small number of characteristic clusters in which cortical network can dynamically operate. These regimes correspond to distinct global brain states and are correlated with the occurrence of major wake-sleep states.

Although our state-space framework obtained encouraging results, future work should address several issues. In particular, we have mainly studied the ability of a state-space representation in tracking global brain dynamics at low temporal resolution and operating within a few clusters, quantitatively inferred by hierarchical clustering algorithms. One difficulty here is the determination of the minimal number of physiologically meaningful clusters. The problem of dimension reduction is very difficult, especially when the target categories for classification remain unknown. It remains possible that other dimension reduction techniques might provide useful physiological features and identify more dynamic states. Especially, future methods could improve upon the poor temporal resolution in the state-space method because of the smoothing procedure. However, given the relatively slow temporal evolution of behavioral states, such slow temporal dynamics are likely well captured by the state-space framework.

How can this description be useful? The state-space framework proposed here may be helpful for sleep stage scoring (i.e., the process of classifying the different stages of the sleep). Indeed, it is known that most of the current stage-coding approaches, both manual and automatic, face several important limitations [15]. Furthermore, our state-space representation may help to better describe transitions between different sleep stages. Indeed, according to the recommendations of Rechtschaffen and Kales [16], most algorithms to identify wake-sleep states based on EEG features implicitly assume that the wake-sleep cycle consists of several categorically different and stable states. This approach tends to characterize the wake-sleep cycle as a stair case process, jumping back and forth between a set of state. This stair case representation of states promotes the unrealistic view that state transitions occur instantaneously, with no intermediate periods between them, even when the dynamics of the system do not clearly resemble any predefined states [9]. Finally, in the context of neurology, disturbances of large-scale synchronized networks have been implicated in several brain disorders, such as epilepsy, schizophrenia, autism, and Parkinson's disease [17]. Our state-space representation of global brain dynamics may help to identify pathological alterations in large-scale patterns. In particular, this representation may be helpful in identifying dynamic state fluctuations of the epileptic brain and possibly characterizing long-term pathological transitions to seizures [12, 18].

Overall, our present results strengthen the recent observations in rats that behavioral states and their transitions can be identified by synchronizations across/within forebrain areas [9]. Additionally, our results support the role of oscillation-mediated temporal links, activated differently depending on the ongoing behavioral state, in the coordination of specific information transfer between distant brain regions [19]. Furthermore, following earlier proposals [20, 21], our descriptions make explicit generic structures of large-scale brain dynamics, that is, characteristics that are observed independently of the particular variation of the network under consideration. Together, these results provide new insights into the neurophysiological correlates of state-dependent aspects of human brain synchronization. We anticipate that this type of comprehensive quantification will have powerful applications in the development of automatic recognition of behavioral states [22].
